# Supplementary material for: The nepenthesin insert in the Plasmodium falciparum aspartic protease plasmepsin V is necessary for enzyme function
Source: J Biol Chem. 2022 Aug 9;298(9):102355. doi: 10.1016/j.jbc.2022.102355 (PMC9478907; doi:10.1016/j.jbc.2022.102355)
Supplement: Supplemental Figures S1–S9 and Table S1 [file mmc1.pdf]

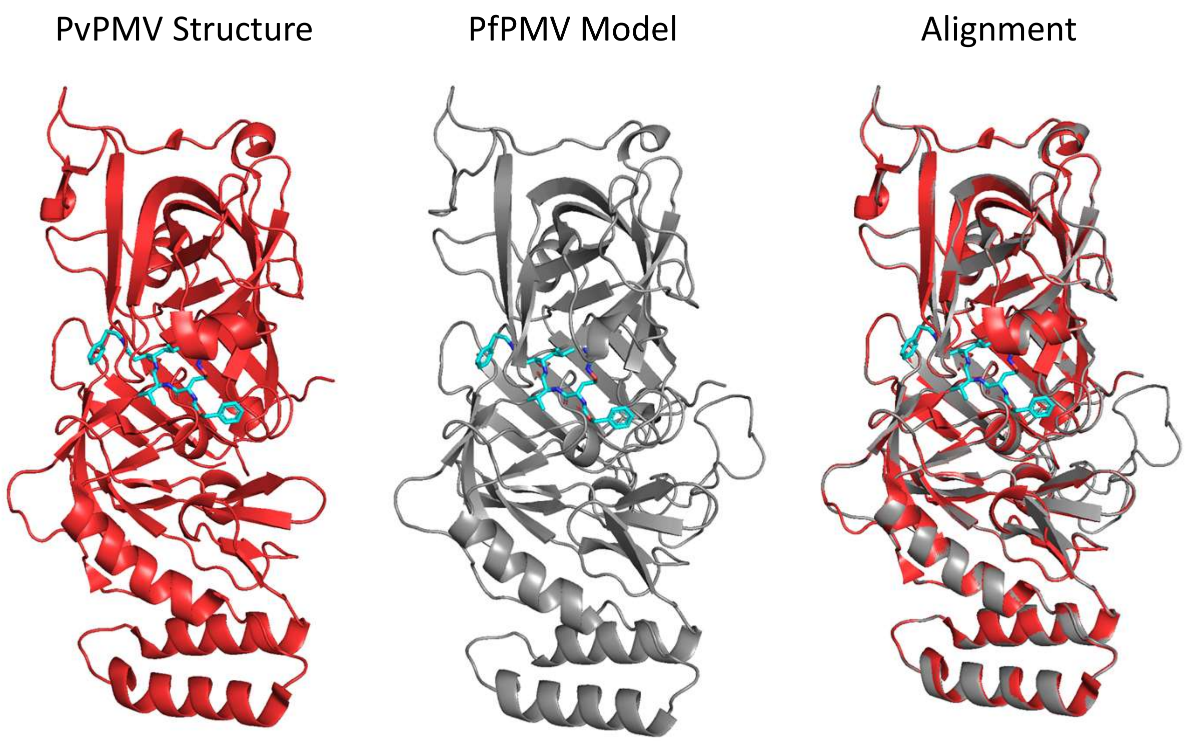

Supplemental Figure 1 – Alignment of the experimentally determined *P. vivax* PM V (PvPMV) structure (left, red) and the *P. falciparum* PM V (PfPMV) model predicted by SWISS-MODEL (grey, center). At right, the two structures are aligned, showing their gross structural similarity. Peptidomimetic inhibitor WEHI-842 is shown in blue.

PfPMV	YNLNYSKTSSILY**C**NKSN**CP**YG**L**K**CV**GNK**CEY**L**QSYCEGS**Q**I**Y**GFYFSD**I**V**TLP**S**YN**N**K-
PvPMV	FNLNNSKTSSILY**C**ENEE**CP**FK**L**N**CV**KGK**CEY**M**QSYCEGS**Q**I**S**GFYFSD**V**V**SVV**S**YN**N**E-
PkPMV	FNLNNSKTSSILY**C**ENEK**CP**YN**L**N**CV**NGK**CEY**L**QSYCEGS**Q**I**S**GFYFSD**V**V**TMT**S**YS**N**E-
PbPMV	FNLNNSSTSSILY**C**NDNI**CP**YN**L**K**CV**KGR**CEY**L**QSYCEGS**R**I**N**GFYFSD**I**V**RLE**S**NN**N**TK
PyPMV	FNLNNSSTSSVLY**C**NDNT**CP**YN**L**K**CV**KGR**CEY**L**QSYCEGS**R**I**N**GFYFSD**V**V**KLE**S**TN**N**TK
PfPMI	FNPNKSRTFTKNLKNNQ-E----------SVYTYIQYGTGTSILEQSYDDVYLK-GLKIK
PfPMII	YKRTKSFVYKYDKKG---L----------PSVIEIFYLSGKIVAFEGYDTIYLGKKLKIP
PfPMIII	YDSSKSKTYEKD-----------------DTPVKLTSKAGTISGIFSKDLVTIG-KLSVP
PfPMIV	YDASASKSYEKD-----------------GTKVEISYGSGTVRGYFSKDVISLG-DLSLP
PfPMVI	FNPNKSRTFTKNLKNNQ-E----------SVYTYIQYGTGTSILEQSYDDVYLK-GLKIK
PfPMVII	YKRTKSFVYKYDKKG---L----------PSVIEIFYLSGKIVAFEGYDTIYLGKKLKIP
PfPMVIII	YDHKISKNYKLVKK---------------KDPVEILFGTGEIHIAYVTDDIHLG-DIKVK
PfPMIX	YNHKLSSSFKYYEP---------------HTNLDIMFGTGIIQGVIGVETFKIG-PFEIK
PfPMX	YDPNKSKTFRRSFI---------------EKNLHIVFGSGSISGSVGTDTFMLG-KHLVR

Supplemental Figure 2 – Sequence alignment of the area around the nepenthesin insert for PM V from several species, as well as all *P. falciparum* plasmepsins. Alignments were performed using Clustal Omega. Residues shared among the PM V sequences are bolded. The nepenthesin insert is highlighted in yellow.

```
PfPMV   ----------------------------MNNYFLRKENFFILFCFVF------VSIFFVSNVTIIKCNNVENK-----ID   41
PvPMV   --------------------------------------------------------------------------------    0
PbPMV   ------------------------------------------MCFSK--LYNFITYFIIINILV-QA-------------
PfPMI   MALSIKEDFSSAFAKNESAVNSSTFNNNMKTWKIQ-KRFQILYVFFFLLITGALFYYLIDNVLFPKNKKINEIMNTSKHV

PfPMV   NVGKKIENVGKKIGDMENKNDN------------VE-NKNDNVGNKNDNVKNASSDLYKYKLYGDIDEYAYYFLDIDIGK  109
PvPMV   MVGASLGPPGRG--SLSRLIRLVICV--------LTLCALSVQGRSESTEGHSKDLLYKYKLYGDIDEYAYYFLDIDIGT   70
PbPMV   ----------------NTEN--------------------EDILN---KNSEKNEEIYKYKLYGDIDEYAYYFMDINIGT
PfPMI   IIGFSIENSHDRIMKTVKQHRLKNYIKESLKFFKTGLTQKPHLGNAGDS-------V----TLNDV-ANVMYYGEAQIGD

                                                 Nepenthesin Insert      Flap
PfPMV   PSQRISLILDTGSSSLSFPCNGCKDCGIHMEKPYNLNYSKTSSILYCNKSNCPYGLKCVGNKCEYLQSYCEGSQIYGFYF  188
PvPMV   PEQRISLILDTGSSSLSFPCAGCKNCGVHMENPFNLNNSKTSSILYCENEECPFKLNCVKGKCEYMQSYCEGSQISGFYF  150
PbPMV   PGQKLSLIVDTGSSSLSFPCSECKDCGVHMENPFNLNNSSTSSILYCNDNICPYNLKCVKGRCEYLQSYCEGSRINGFYF
PfPMI   NKQKFAFIFDTGSANLWVPSAQCNTIGCKTKNLYDSNKSKTYEK------------DGTKVEM----NY-VSGTVSGFFS

PfPMV   SDIVTLPSYNNK--NKISFEKLMGCHMHEESLFLHQQATGVLGFSLTKP---NGVPTFVDLLFKHTPSL-KPI---YSIC  259
PvPMV   SDVVSVVSYNNE---RVTFRKLMGCHMHEESLFLYQQATGVLGMSLSKP---QGIPTFVNLLFDNAPQL-KQV---FTIC  220
PbPMV   SDIVRLESNNNTKNGNITFKKHMGCHMHEEGLFLHQHATGVLGLSLTKP---KGVPTFIDLLFKSSPKL-NKI---FSLC
PfPMI   KDIVTIANLSFP----YKFIEVTDTNGF-EPAYTLGQFDGIVGLGWKDLSIGSVDPVVVEL--KNQNKIEQAVFTFYLPF

                                     Unstructured region
PfPMV   VSEHGGELIIGGYEPDYFLSNQKEKQKMDKSDNNSSNKGNVSIKLKNNDKNDDEENNSKDVIVSNNVEDIVWQAITRKYY  339
PvPMV   ISENGGELIAGGYDPAYIVRRGGSKSVSGQ------GSGPVSESLS--------ESGEDPQVALREAEKVVWENVTRKYY  286
PbPMV   ISEYGGELILGGYSKDYIVKEVSIDEKKDNIEH--------------------NKNENINSINKSIVDGILWEAITRKYY
PfPMI   DDKHKGYLTIGGIEDRFYEGQLT-----------------------------------------------YEKLNHDLY

                                                                     Helix-turn-helix
PfPMV   YYIKIYGLDL-YGTNIM-DKKELDMLVDSGSTFTHIPENIYNQINYYLDILCIHDMTNIYEINKRLKLTNESLNKPLVYF  417
PvPMV   YYIKVRGLDM-FGTNMMSSSKGLEMLVDSGSTFTHIPEDLYNKLNYFFDILCIQDMNNAYDVNKRLKMTNESFNNPLVQF  365
PbPMV   YYIRVKGFQL-FGTTFSHNNKSMEMLVDSGSTFTHLPDDLYNNLNFFFDILCIHNMNNPIDIEKKLKITNETLSNHLLYF
PfPMI   WQVD---LDLHFGNL---TVEKATAIVDSGTSSITAPTEFLNKFFEGLDVVKIPFLP----------LYITTCNNPKLPT

PfPMV   EDFKTALKNIIQNENLCIKIVDGVQCWKSLENLPNLYITLSNNYKMIWKPSSYLYKKESF---WCKG--LE-KQVNNKPI  491
PvPMV   DDFRKSLKSIIAKENMCVKIVDGVQCWKYLEGLPDLFVTLSNNYKMKWQPHSYLYKKESF---WCKG--IE-KQVNNKPI  439
PbPMV   DDFKSTLKNIISSENVCVKIADNVQCWRYLENLPNIYIKLSNNTKLVWQPSSYLYKKESF---WCKG--LE-KQVNDKPI
PfPMI   LEFRSATNVY-----------------------------------TLEPEYYLQQIFDFGISLCMVSIIPVDLNKNTFI

PfPMV   LGLTFFKNKQVIFDLQQNQIAFIESKCPSNLTSSRPRTFNEYREKENIFLKVSYINLYCLWLLLALTILLSLILYVRKMF  571
PvPMV   LGLTFFKNRQVIFDIQKNRIGFVDANCPSHPTHTRPRTYNEYKRKDNIFLKIPFFYLYSLFVVFALSVLLSLVFYVRRLY  519
PbPMV   LGLSFFKNKQIIFDLKNNKIGFIESNCPSNPINTRPRTFNEYNIKENHLFKQSYFSLYAFSIIIALTFILYIILYIKKFI
PfPMI   LGDPFMRKYFTVFDYDNHTVGFALAKKKL---------------------------------------------------

PfPMV   YMDYFPLSDQNKSPIQEST   590
PvPMV   HMEYSPLPSEGKAPADA--   536
PbPMV   CSYYNPLHG----------
PfPMI   -------------------
```

Supplemental Figure 3 – Sequence alignment of the reference sequences for *P. falciparum*, *P. vivax*, and *P. berghei* PM V, as well as *P. falciparum* plasmepsin I (PfPMI). Alignments were performed using Clustal Omega. Residues shared among the PM V sequences are bolded. Structures of interest are highlighted.

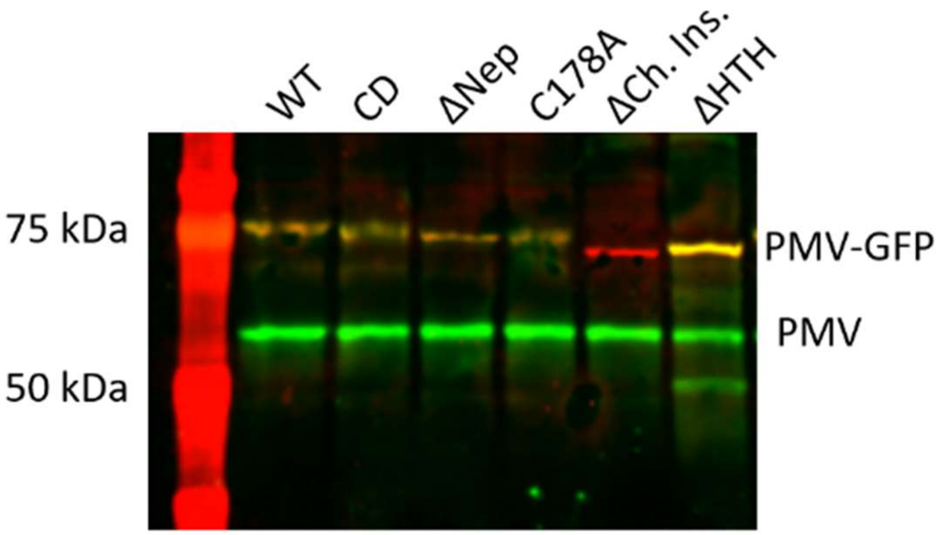

Supplemental Figure 4 – Western blot with anti-PMV (green) and anti-GFP (red) showing that mutants enzymes can be detected by monoclonal anti-PMV or anti-GFP, with the exception of the ΔCh. Ins. mutant, which is no longer recognized by anti-PMV.

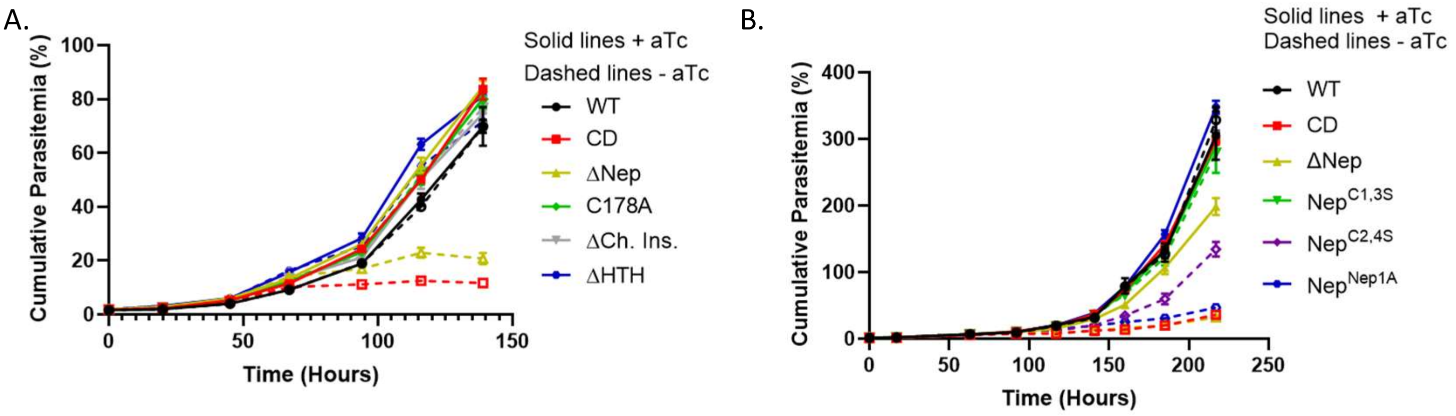

Supplemental Figure 5 – Full growth curves for Fig. 2D and Fig. 3D. Knockdown was performed as described above and parasite growth measured daily by flow cytometry. All parasites were subcultured 1:2 any time a well's parasitemias grew above 5%. Reads were then multiplied appropriately to calculate "cumulative parasitemias".

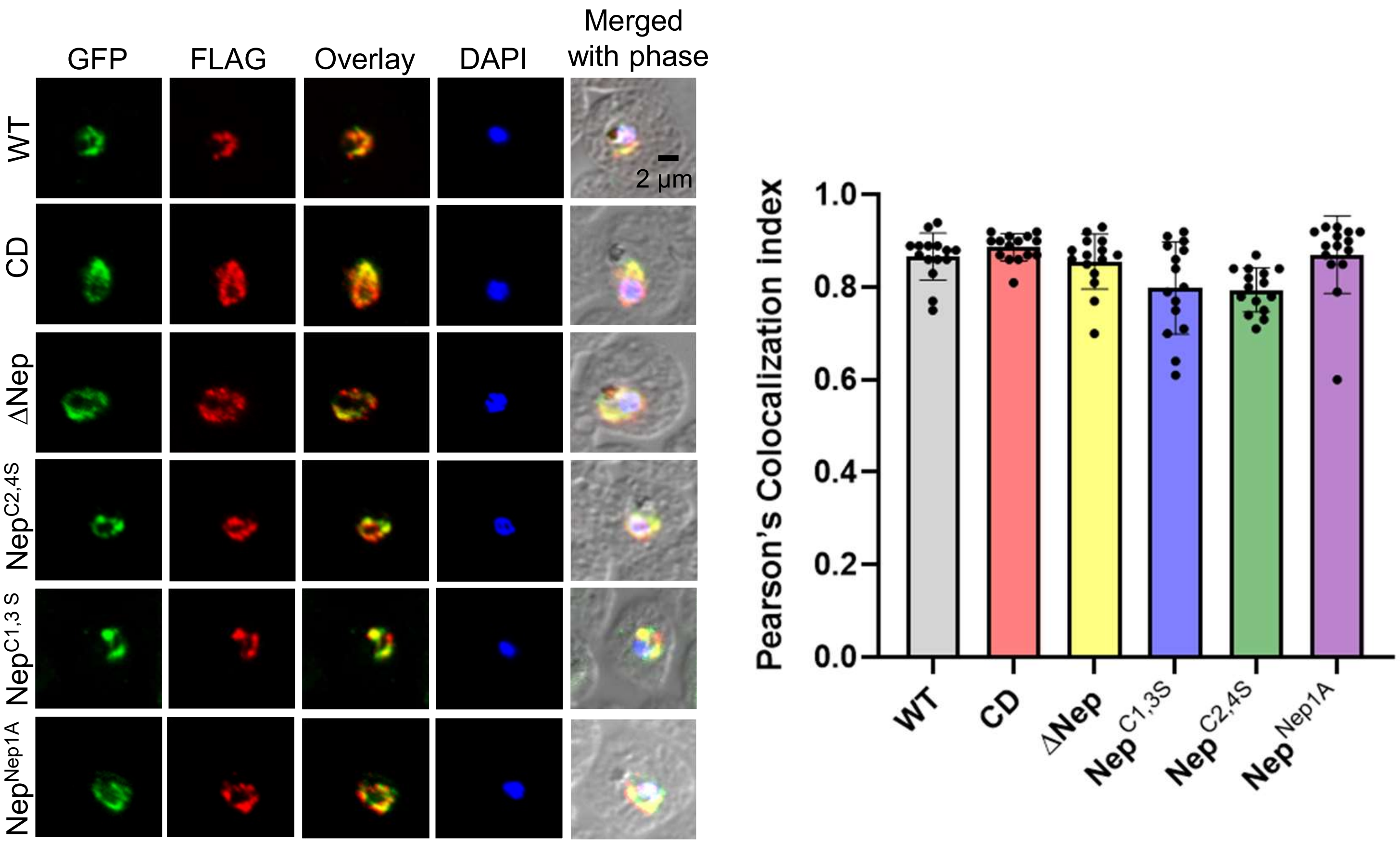

Supplemental Figure 6 – Nepenthesin insert mutant PM V colocalizes with endogenous PM V - Ring-stage parasites (15-20 hours post-invasion) were fixed and stained with anti-GFP (for the mutant enzymes) and anti-FLAG (the endogenous enzyme). Correlation was analyzed via Pearson's Correlation Coefficient, using 15 parasites per mutant. Individual data points are shown, as are the mean of the 15; error bars represent the standard deviation of the measurements.

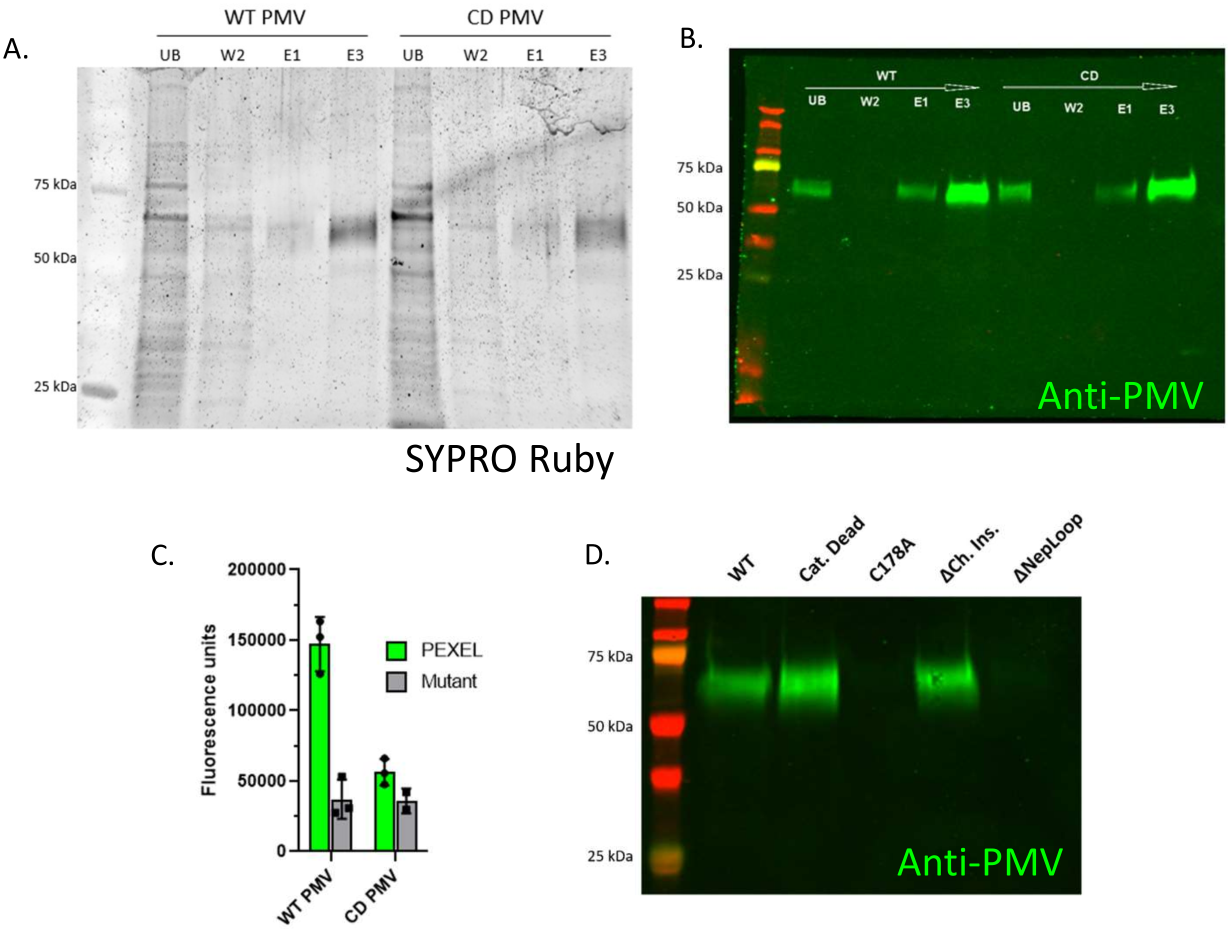

Supplemental Figure 7 – Inconsistent expression of PM V mutants in HEK293 cells (A) SYPRO Ruby-stained gel showing purification scheme for secreted WT and CD PM V from HEK293 conditioned media. Fractions shown are unbound (UB), a wash fraction (W2), and two elution fractions (E1 and E3). (B) Western blot of the same samples probed with anti-PM V. (C) Activity assay for WT and CD PM V showing recombinant PM V to be active against a PEXEL peptide but not the mutant peptide. Assay was performed as in Fig. 5B. (D) Western blot probed with anti-PM V.

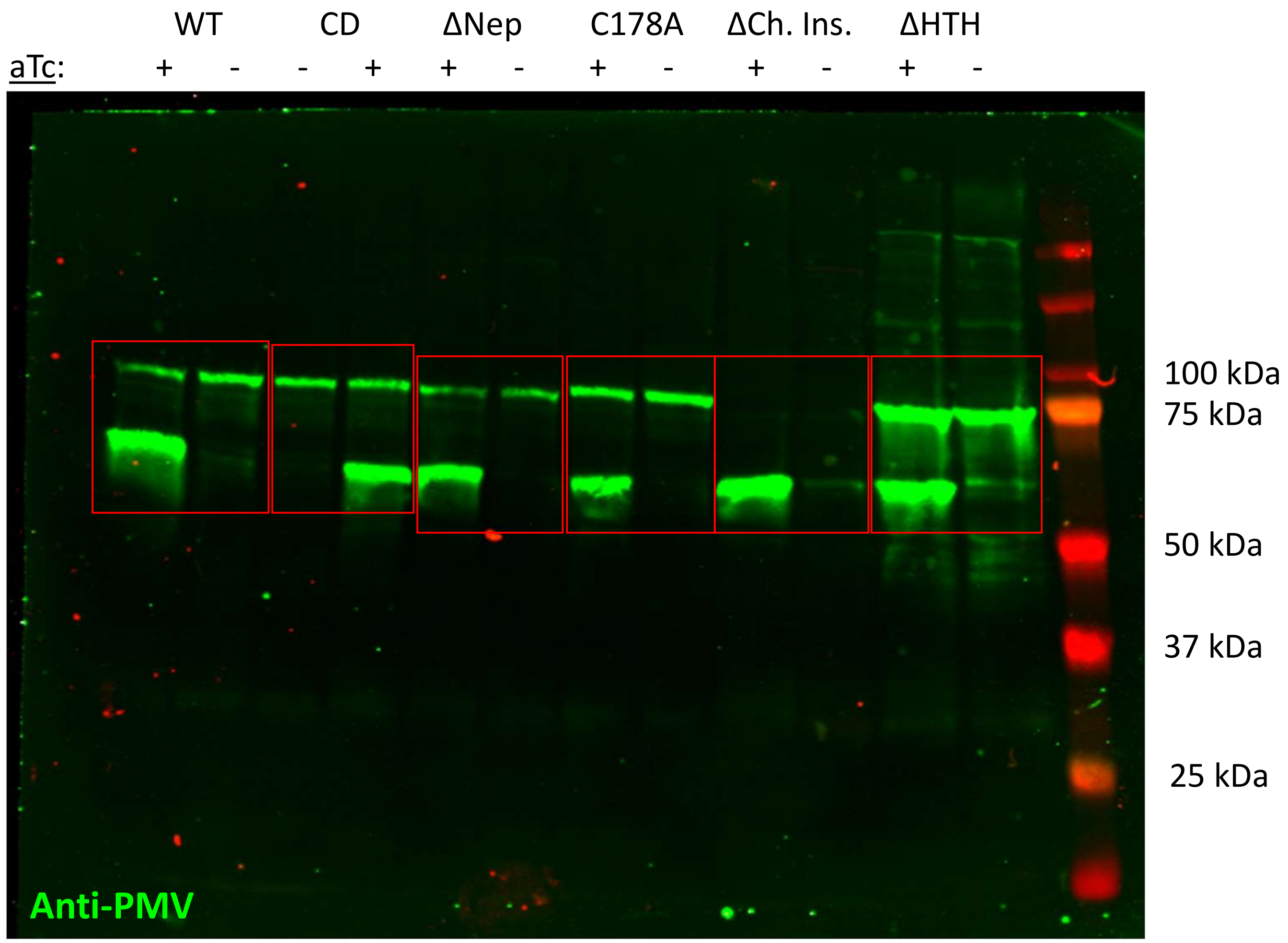

Supplemental Figure 8 – Uncut gel used to generate Fig. 2C. Pairs of lanes were cropped to facilitate visual comparison; crop/splice sites are marked with red boxes. The cropped image of lanes 3 & 4 was horizontally flipped so that the lane order would match the other mutants to aide the reader's eye. The ΔHTH mutant is driven by a stronger promoter than the others (see Results: "Regulatable system for PM V depletion and rescue"); brightness was adjusted to match the other mutants for display in Fig. 2.

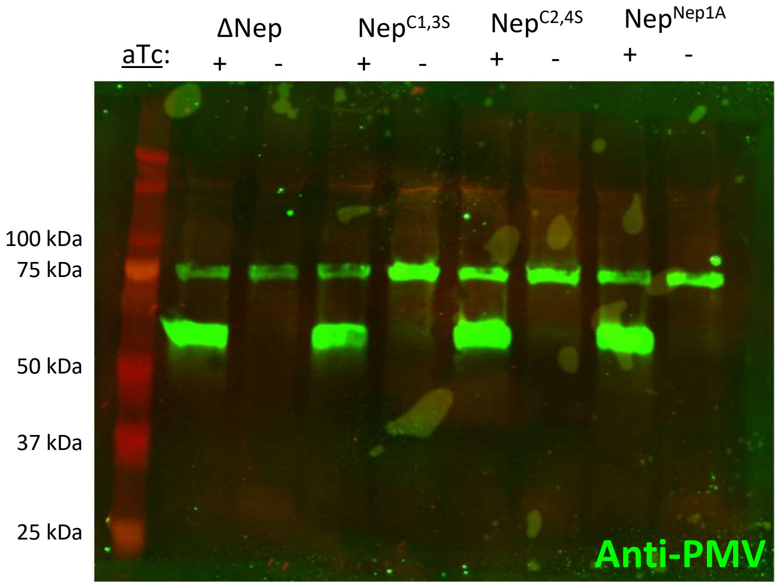

Supplemental Figure 9 – Uncut gel used to generate Fig. 3C.

**Name** (use)	**Sequence**
**1** (attB into EOE)	GGTCGACTCTAGAGGATCCCCGGGTACCGAGCTCGAATTCTGGTTTGTCTGGTCAACCACCGCGGTCTCAGTGGTGTACGGTACAAACCCGAATTCTGGTTTGTCTGGTCAACCACCGCGGTCTCAGTGGTGTACGGTACAAACCCGGAATTCTAGATTTAATAAATATGTTCTTATATATAATG
**2** (PM V FWD)	GTGCCACCTGACGTCGAGGTGGGTGGAGGGGTTTTATCCATGAGAGG
**3** (PM V REV)	CTGCACCTGGcctaggTGTTGATTCCTGTATGGGAG
**4** (PM V FWD HTH)	ACGATTTTTTctcgagATGAATAATTATTTTTTAAGGAAAGAAAATTTTTTTATATTG
**5** (CD)	CCATCGCAAAGAATTTCTTTAATTCTAGcTACAGGTTCATCTTCGTTAAGTTTCCCGTG
**6** (ΔNep)	AAACATCATCTATTTTATATGAATATCTTCAATCGTATTG
**7** (C178A)	GTGAATATCTTCAATCGTATgctGAAGGGTCTCAAATATATGG
**8** (ΔCh. Ins.)	GTTATGAACCGGATTACTTCGATATTGTGTGGCAAGCTAT
**9** (ΔHTH)	CCAAATAAATTATTATTTAGATATTTTATGTATACATGATATGgccgccAATTTATGTATTAAAATAGTTGATGGAGTACAATGTTGG
**10** (Nep^{C1,3S})	CAAAAACATCATCTATTTTATATaGTAATAAATCCAATTGTCCTTATGGTTTAAAAaGTGTAGGAAATAAATGTGAATATC
**11** (Nep^{C2,4S})	CATCTATTTTATATTGTAATAAATCCAATaGTCCTTATGGTTTAAAATGTGTAGGAAATAAAaGTGAATATCTTCAATCGTATTGTG
**12** (Nep^{Nep1A})	GAATTATTCAAAAACATCATCTATTTTATATTGCTCAAGCCAACTCTGTCAAGCCCTTTCAAGCCCGACATGCTCTAATAATTTCTGCGAATATCTTCAATCGTATTGTGAAGGG
**13** (Seq 1)	GGTATTCATATGGAAAAACCATATAACTTG
**14** (Seq 2)	CACATATTCCAGAAAATATTTATAACC
**15** (Seq 3)	GTGGAAAATAAAAATGACAATGTGGGAAATAAAAATGACAATG
**16** (Seq 4)	CGTTAAGTTTCCCGTGTAATGGTTG
**17** (PMV into pHL)	GCGTAGCTGAAACCGGTgtaGAGAATAAAATCGATAATGTTGG
**18** (PMV into pHL)	GATGGTGGTGCTTGGTACCTGACGGGCACTTGC
**19** (pHL CD)	CATCCCAGCGTATATCTTTGATCTTAGcTACCGGTAGCAGCTCTTTGTCTTTTCC
**20** (pHL ΔNep)	CTACTCAAAAACAAGTTCTATTCTTTAtgAATACCTACAAAGTTATTGTGAAGGAAG
**21** (pHL C178A)	GGAAATAAATGTGAATACCTACAAAGTTATgctGAAGGAAGTCAAATATATGGTTTC
**22** (pHL ΔCh. Ins.)	CATCGGCGGTTATGAACCCGATTACTTcgATATTGTATGGCAAGCAATAACAAGG
**23** (pHL ΔHTH)	GGATATTTTGTGCATACATGACATGgccgccAACTTGTGTATAAAAATAGTTGATGG
**24** (pHL seq 1)	GACATAGGAAAACCATCCCAGCG
**25** (pHL seq 2)	GGTAGCACTTTCACGCATATACCTG

Supplemental Table 1 – Primers used in this study.
